# Are “Ultraconserved” Genetic Elements Really Indispensable?

**DOI:** 10.1371/journal.pbio.0050253

**Published:** 2007-09-04

**Authors:** Liza Gross

With over 180 genomes sequenced to date and counting, researchers must rely on certain assumptions to help them sift through mountains of data and identify the most promising candidates for functional analysis. One of the guiding principles of comparative genome analysis assumes that highly conserved DNA sequences—which show little variation across species—have been preserved throughout evolution because they encompass important biological functions.

In 2004, researchers identified a unique category of long sequences (spanning at least 200 DNA base pairs) in the human genome that are exactly the same in the mouse and rat. Though over half of these “ultraconserved” genetic elements don’t code for gene products, their concentration near coding regions (for transcription factors and molecules involved in developmental processes), along with some experimental evidence, suggests that they may play a role in gene regulation. The discovery of ultraconserved sequences stimulated vigorous debate about the mechanisms that may have led to such mutational restraint. It also provided an unprecedented opportunity to test the conventional wisdom that these sequences encode fundamental functions—how else to explain their perfect preservation over the 80 million or so years since the rodent and primate lineages diverged?

In a new study, researchers at the Joint Genome Institute and Lawrence Berkeley National Laboratory used standard transgenic techniques to test these long-held assumptions, with unexpected results. Nadav Ahituv, Len Pennacchio, Edward Rubin, and colleagues reasoned that if ultraconserved elements are as vital as predicted by theory, then deleting them from an animal should cause severe abnormalities that result in infertility or death. To their surprise, the researchers found that all of the mice tested not only survived these expected lethal deletions but did so with no apparent observable effect (or phenotype).

The researchers increased the probability of seeing an effect by carefully choosing the elements for deletion. The elements had to not only function as enhancers (that is, promote transcription) when inserted near a reporter gene in transgenic mice, but also reside near genes that produce profound phenotypes when disturbed due to mutations. After identifying four elements that met these criteria—uc248, uc329, uc467, and uc482—the researchers engineered “knockout” strains of mice that lacked one of the four elements.

The transgenic strains all survived and reproduced as expected. Their offspring showed no appreciable differences in viability or litter size compared to their control (wild-type) littermates and no anomalies in body weight, age, or survival. The researchers also ran standard clinical chemistry tests for signs of disease, as well as expression analyses of the genes near each element; these tests revealed only modest differences between the transgenic and wild-type offspring.

**Figure:**
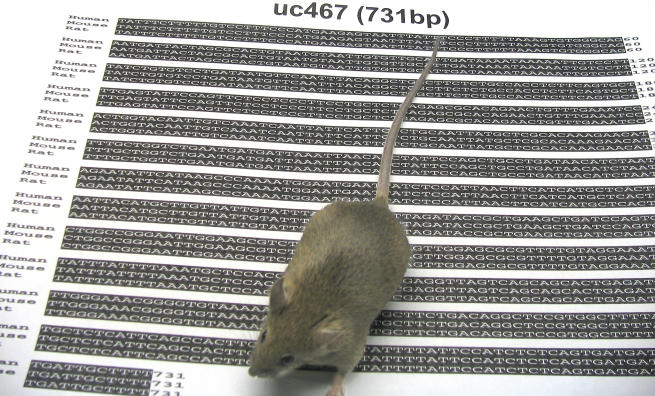
The sequence beneath the female mouse—a six-month-old homozygous knockout for “ultraconserved” element 467—which was deleted from her genome, shows the perfect preservation over 731 base pairs between the human, mouse and rat.

In addition to these molecular screens, the researchers also examined each of the lines for physical defects associated with aberrant expression of genes near the deleted elements. For example, the uc248 element is flanked by genes that code for two transcription factors, *DMRT1* and *DMRT3*. Though it’s known that mice born without either copy of *DMRT1* develop testicular defects, the researchers had to generate their own knockout strain of *DMRT3* mice to identify the phenotype associated with ablating the gene. Offspring developed such severe misalignment of their teeth that they died of starvation within two months, and some males developed sexual abnormalities. Transgenic mice lacking the corresponding ultraconserved element, uc248, showed no signs of similar sexual or dental problems.

Mutations in genes near each of the other ultraconserved elements revealed a range of similarly lethal or severe abnormalities, ranging from neurological and sexual disorders to defective eye and kidney development. But in no case did the researchers find comparable aberrations in mice lacking the adjacent ultraconserved elements.

These results challenge the prevailing notion that highly conserved elements necessarily encode essential functions. Still, the researchers acknowledge that their experimental setup could have missed phenotypic changes that may have emerged under other conditions (in the wild, for example, or over multiple generations). Since all the ultraconserved elements were chosen based on their ability to promote transcription in lab tests—ensuring that the elements were capable of function—it’s possible that deleting them produced no obvious effects because other elements stepped in to perform their job. Future studies can explore these possibilities and continue to probe the mechanisms that gave rise to such extreme evolutionary conservation. But for researchers relying on sequence constraint to shed light on the function of billions of noncoding base pairs in the human genome, the question remains: Why would evolution preserve these noncoding elements if their loss has no significant effect on the viability, fertility, and function of the organism?

